# Inference of gene networks using gene expression data with applications

**DOI:** 10.1016/j.heliyon.2024.e26065

**Published:** 2024-02-09

**Authors:** Chi-Kan Chen

**Affiliations:** Department of Applied Mathematics, National Chung Hsing University, 145 Xingda Rd., South Dist., Taichung City, 40227, Taiwan

**Keywords:** ARD, Cancer, Co-expression, Gene expression, Hub, Inference, Network, Partial correlation

## Abstract

Gene networks (GNs) use graphs to represent the interaction relationships between genes. Large-scale GNs are often sparse and contain hub genes that interact with many other genes. In this paper, we propose a novel method called NetARD, which utilizes Automatic Relevance Determination (ARD) to estimate partial correlations, to infer GNs with the hub genes from gene expression data. We test NetARD on simulated GNs and in silico GNs, and it outperforms existing methods. In our high-throughput gene expression data analysis, we integrate the NetARD into a method called GN Co-expression Extension (GNCE). This approach infers the GNs of co-expressed genes, with genes from a predefined GN serving as hub genes. We validate this approach by extending the core GN of transcription factor genes of *E. coli* using microarray data. In an application example, we identify biological process (BP) Gene Ontology (GO) terms that are significantly involved in cancer progression. This task is accomplished by analyzing the GN inferred through GNCE using the core GN associated with the colorectal cancer pathway and RNA-seq data.

## Highlights

• The NetARD uses the ARD to estimate partial correlations between genes to infer GNs with hubs from gene expression data.
• When tested on simulated and in silico GNs, the NetARD method demonstrates superior performance compared to existing methods.
• The GNCE uses the NetARD to infer GNs for co-expressed genes using genes from a pre-defined core GN as hub genes.
• Validation of the GNCE involves extending the core GN of transcription factor genes in *E. coli* using microarray data.
• The GNCE is applied to extend the predefined core GN to identify GO terms related to colorectal cancer using RNA-seq data.

## 1 Introduction

Genes interact with each other through gene expression products in a living cell. The gene network (GN) represents the interaction relationships between genes by a graph whose nodes correspond to genes and undirected and directed edges represent the interactions and causal regulatory relationships between genes, respectively. The identification of GNs is crucial for gaining insights into cellular functions and disease-associated biological processes. The results obtained from studying GNs have important applications in the fields of cellular biology and medicine [1]. With the advancements in DNA microarray and RNA-seq technologies, it is now possible to measure the mRNA expression levels of thousands of genes in an experiment, thereby capturing information about gene interactions. Mathematical or statistical networks have been employed as models to infer GNs using gene expression data. For a comprehensive understanding of model-based GN inference methods, one can refer to reviews such as [[2], [3], [4]].

GNs involved in biological processes often demonstrate sparsity, where individual genes typically interact with only a limited number of other genes. However, within these GNs, there are also hub genes that exhibit extensive interactions with a large number of genes. A prime example of such hub genes is those responsible for encoding transcription factors (TFs), which have the capability to regulate the expression of numerous genes through their TF products. Also, it has been noted that large-scale GNs often exhibit hub genes in the presence of scale-free topology [5]. Hub genes, which are central to these GNs, play a critical role in cell survival and are potential targets for drug development. In recent years, network model-based methods have been developed to infer GNs with hub genes or scale-free topology from high-throughput gene expression data, e.g., correlation and weighted correlation networks [6,7], partial correlation networks or Gaussian graphical models (GGMs) [8,9], and vector autoregressions and regression-based dynamic Bayesian networks [[10], [11], [12]]. These methods employ diverse regularization techniques to incorporate desired topological properties into the inferred GNs. However, inferring large-scale GNs still poses a challenging problem due to the intricate nature of gene interactions. With a vast number of genes potentially interacting directly or indirectly, unraveling these complex interactions become increasingly difficult.

In this paper, we utilize Automatic Relevance Determination (ARD) [13], a class of sparse Bayesian regressions. Our approach, called NetARD, utilizing the ARD to estimate partial correlations, enables us to infer gene networks (GNs) with hub genes from gene expression data. To validate the NetARD, we compare its performance against published methods in the inference of small-scale simulated GNs featuring hub genes, as well as in silico GNs. In our analysis of high-throughput gene expression data, we begin with a predefined core GN. We extend this core GN using the Gene Network Co-expression Extension (GNCE) method. This method incorporates co-expressed genes that interact with the core GN genes. In this process, we utilize the NetARD to infer the GNs of co-expressed genes, with genes from a predefined GN serving as hub genes. To evaluate the efficiency of GNCE, we implement it on microarray data to extend the transcription factor GN of *E. coli*. In our application example, we identify the Gene Ontology (GO) terms significantly associated with the colorectal cancer progression. This task is achieved by analyzing the GN that is extended via the CNCE from the core GN associated with the colorectal cancer pathway using the RNA-seq data.

## 2 Methods

### 2.1 Partial correlation network model

Let us consider the continuous random vector $X={\left(X_{1},\cdots ,X_{p}\right)}^{T}\in R^{p\times 1}$ that contains the expression levels of genes 1,⋯,p. We assume that the mean vector $E\left(X\right)=0_{p\times 1}\in R^{p\times 1}$ and the covariance matrix $\text{Cov}\left(X\right)=\Sigma \in R^{p\times p}$, where Σ is positive-definite, denoted as Σ≻0. Let us denote by $X^{\backslash k^{\prime }k}\in R^{\left(p-2\right)\times 1}$ the subvector of X that excludes $X_{k^{\prime }}$, $X_{k}$ $\left(k^{\prime }\neq k\right)$. Assume $\sum_{j\neq k^{\prime },k}c_{j}^{\left(k\right)}X_{j}$ represent the optimal linear regression with the minimum variance $\text{Var}\left(\varepsilon_{k^{\prime }}^{\left(k\right)}\right)$, where $\varepsilon_{k^{\prime }}^{\left(k\right)}=X_{k}-\sum_{j\neq k^{\prime },k}c_{j}^{\left(k\right)}X_{j}$. This linear regression is selected from all possible linear regressions of $X_{k}$ on $X^{\backslash k^{\prime }k}$. The correlation $\pi_{k^{\prime }k}=\text{Corr}\left(\varepsilon_{k}^{\left(k^{\prime }\right)},\varepsilon_{k^{\prime }}^{\left(k\right)}\right)\in \left[-1,1\right]$ defines the partial correlation between $X_{k^{\prime }}$, $X_{k}$. It reveals the direct association between $X_{k^{\prime }}$, $X_{k}$ after accounting for the effects of genes in $X^{\backslash k^{\prime }k}$. Let us denote by $\omega_{k^{\prime }k}$ the $k^{\prime },k$ entry of precision matrix $\Omega =\Sigma^{-1}$. It holds that

(1) $$\pi_{k^{\prime }k}=-\frac{\omega_{k^{\prime }k}}{\sqrt{\omega_{k^{\prime }k^{\prime }}\omega_{kk}}}\;\left(k^{\prime }\neq k\right)\text{.}$$

Moreover, assume $\sum_{j\neq k}b_{j}^{\left(k\right)}X_{j}$ is the optimal linear regression of $X_{k}$ on $X^{\backslash k}$. It holds that

(2) $$b_{k^{\prime }}^{\left(k\right)}=-\frac{\omega_{k^{\prime }k}}{\omega_{kk}}\;\left(k^{\prime }\neq k\right)\text{.}$$

Both Eqs. (1), (2) can be verified by utilizing the partitions of Σ and Ω, along with the formulas for optimal regression coefficient vectors expressed in terms of submatrices of Σ. For instance, a reference for the derivation of Eq. (2) can be found in Eqs. (17.17)–(17.9) of [14]. It follows that

(3) $$\pi_{k^{\prime }k}=\text{sign}\left(b_{k^{\prime }}^{\left(k\right)}\right)\;\sqrt{b_{k^{\prime }}^{\left(k\right)}b_{k}^{\left(k^{\prime }\right)}}\;\left(k^{\prime }\neq k\right)\text{.}$$

Let the undirected graph G=(V,E) be a representation of the GN, where V contains nodes corresponding to $X_{k}$ (k=1,⋯,p) and E⊆V×V comprises undirected edges connecting nodes in V. The edge $\left(k^{\prime },k\right)$ connecting nodes $k^{\prime }$, k signifies the interaction between gene $k^{\prime }$ and gene k. Let $W\in R^{p\times p}$ be the weighted adjacency matrix of G, where the $k^{\prime },k$ entry $w_{k^{\prime }k}=\left|\pi_{k^{\prime }k}\right|\in \left[0,1\right]$ if $k^{\prime }\neq k$ and 0 if $k^{\prime }=k$. An edge $\left(k^{\prime },k\right)\in E$ if $w_{k^{\prime }k}>0$, ∉E if $w_{k^{\prime }k}=0$. Moreover, if $X∼N_{p}\left(0_{p\times 1},\Sigma \right)$ then $w_{k^{\prime }k}>0$ if and only if $X_{k^{\prime }}$, $X_{k}$ are conditionally dependent given $X^{\backslash k^{\prime },k}$. G represents the structure of GGM in which $\left(k^{\prime },k\right)\in E$ is often interpreted as the direct interaction between gene $k^{\prime }$ and gene k. Gene $k^{\prime }$ is a regulator of gene k and/or vice versa.

### 2.2 p-node ARD

Assume that $X=\left(X^{\left(1\right)},\cdots ,X^{\left(p\right)}\right)\in R^{N\times p}$ is the data matrix that contains N i.i.d. samples of X. Let us denote by $X^{\backslash k}\in R^{N\times \left(p-1\right)}$ the submatrix of X excluding the column $X^{\left(k\right)}$. The linear regression of $X^{\left(k\right)}$ on $X^{\backslash k}$ is given as $X^{\backslash k}b^{\left(k\right)}$, where $b^{\left(k\right)}={\left(b_{k^{\prime }}^{\left(k\right)}{|}_{k^{\prime }\neq k}\right)}^{T}\in R^{\left(p-1\right)\times 1}$. Let $\varepsilon_{k}=X^{\left(k\right)}-X^{\backslash k}b^{\left(k\right)}$. Assume that $P_{k}\left(\varepsilon_{k}|b^{\left(k\right)},\beta \right)∼N_{N}\left(0_{N\times 1},\beta^{-1}I_{N\times N}\right)$, where β>0, $I_{N\times N}\in R^{N\times N}$ is the identity matrix. The likelihood function of $b^{\left(k\right)}$, β given X is defined as $L_{k}\left(b^{\left(k\right)},\beta |X\right)=P_{k}\left(\varepsilon_{k}|b^{\left(k\right)},\beta \right)$. In the Bayesian estimation framework, the ARD assumes that $b^{\left(k\right)}$ follows a 2-level prior [13]. At the first level, the prior $P_{k}\left(b^{\left(k\right)}|\alpha^{\left(k\right)}\right)∼N_{p-1}\left(0_{\left(p-1\right)\times 1},D_{\alpha^{\left(k\right)}}^{-1}\right)$, where $D_{\alpha^{\left(k\right)}}\in R^{\left(p-1\right)\times \left(p-1\right)}$ is a diagonal matrix with $\alpha^{\left(k\right)}={\left(\alpha_{k^{\prime }}^{\left(k\right)}{|}_{k^{\prime }\neq k}\right)}^{T}\in R_{+}^{\left(p-1\right)\times 1}$ on the diagonal. At the second level, the prior $P_{k}\left(\alpha^{\left(k\right)}|s^{\left(k\right)},r^{\left(k\right)}\right)=\prod_{k^{\prime }\neq k}g\left(\alpha_{k^{\prime }}^{\left(k\right)}|s_{k^{\prime }}^{\left(k\right)},r_{k^{\prime }}^{\left(k\right)}\right)$, where $s^{\left(k\right)}={\left(s_{k^{\prime }}^{\left(k\right)}{|}_{k^{\prime }\neq k}\right)}^{T}$ and $r^{\left(k\right)}={\left(r_{k^{\prime }}^{\left(k\right)}{|}_{k^{\prime }\neq k}\right)}^{T}\in R_{+}^{\left(p-1\right)\times 1}$, and $g\left(t|s,r\right)=\frac{r^{s}}{\Gamma \left(s\right)}t^{s-1}e^{-rt}$ (t>0) is the Gamma probability density function. Additionally, we assume the prior $P\left(\beta |s_{\beta },r_{\beta }\right)=g\left(\beta |s_{\beta },r_{\beta }\right)$. With all has been assumed, we formulate pARD as follows.

From the rule of conditional probability, it follows that

(4) $$P_{k}\left(b^{\left(k\right)}|\alpha^{\left(k\right)}\right)L_{k}\left(b^{\left(k\right)},\beta |X\right)=P_{k}\left(b^{\left(k\right)}|X,\alpha^{\left(k\right)},\beta \right)L_{k}\left(\alpha^{\left(k\right)},\beta |X\right)\text{.}$$

Here, $P_{k}\left(b^{\left(k\right)}|X,\alpha^{\left(k\right)},\beta \right)$ represents the conditional posterior probability density function of $b^{\left(k\right)}$ and $L_{k}\left(\alpha^{\left(k\right)},\beta |X\right)$ the marginal likelihood function of regression model specified by $\alpha^{\left(k\right)}$, β. The conditional posterior probability density function $P_{k}\left(\alpha^{\left(k\right)},\beta |X,s^{\left(k\right)},r^{\left(k\right)},s_{\beta },r_{\beta }\right)$ of regression model specified by $\alpha^{\left(k\right)}$, β is proportional to the product of $L_{k}\left(\alpha^{\left(k\right)},\beta |X\right)$ and Gamma priors over $\alpha^{\left(k\right)}$, β. By combining p ARDs, the p-node ARD (pARD) solves the maximization problem of the objective function

(5) $$f\left(\alpha^{\left(1\right)},\cdots ,\alpha^{\left(p\right)},\beta \right)=\sum_{k=1}^{p}\left\{\ln\;L_{k}\left(\alpha^{\left(k\right)},\beta |X\right)+\ln\;P_{k}\left(\alpha^{\left(k\right)}|s^{\left(k\right)},r^{\left(k\right)}\right)+\ln\;P\left(\beta |s_{\beta },r_{\beta }\right)\right\}\text{.}$$

Namely, for each fixed $\Theta =\left(s^{\left(1\right)},r^{\left(1\right)},\cdots ,s^{\left(p\right)},r^{\left(p\right)},s_{\beta },r_{\beta }\right)$, the optimization seeks for the mode $\left({\tilde{\alpha }}^{\left(1\right)},\cdots {,\tilde{\alpha }}^{\left(p\right)},\tilde{\beta }\right)$ of the joint conditional posterior distribution $\prod_{k=1}^{p}P_{k}\left(\alpha^{\left(k\right)},\beta |X,s^{\left(k\right)},r^{\left(k\right)},s_{\beta },r_{\beta }\right)$. The solution to the pARD determines a set of Bayesian regression models. According to Eq. (A1) in Appendix, $P_{k}\left(b^{\left(k\right)}|X,{\tilde{\alpha }}^{\left(k\right)},\tilde{\beta }\right)$ follows the multi-normal distribution and its mean vector is given by

(6) $${\tilde{b}}^{\left(k\right)}={\left({X^{\backslash k}}^{T}X^{\backslash k}+D_{{\tilde{\alpha }}^{\left(k\right)}/\tilde{\beta }}\right)}^{-1}{X^{\backslash k}}^{T}X^{\left(k\right)}\text{.}$$

This mean vector represents the Bayes estimate of $b^{\left(k\right)}$ that is the same as the weighted ridge estimate of $b^{\left(k\right)}$ with the regularization weights in $\frac{{\tilde{\alpha }}^{\left(k\right)}}{\tilde{\beta }}$ for k=1,⋯,p. Algorithm pARD for generating ${\tilde{\alpha }}^{\left(1\right)},\cdots {,\tilde{\alpha }}^{\left(p\right)}$, $\tilde{\beta }$, ${\tilde{b}}^{\left(1\right)},\cdots ,{\tilde{b}}^{\left(p\right)}$ simultaneously is described in Section A1 in Appendix.

For $k^{\prime }\neq k$, we compute the plug-in estimate ${\tilde{\pi }}_{k^{\prime }k}$ of $\pi_{k^{\prime }k}$ using ${\tilde{b}}_{k^{\prime }}^{\left(k\right)}$, ${\tilde{b}}_{k}^{\left(k^{\prime }\right)}$ and Eq. (3). As $0\leq {\tilde{b}}_{k^{\prime }}^{\left(k\right)}{\tilde{b}}_{k}^{\left(k^{\prime }\right)}\leq 1$ is not guaranteed, we define ${\tilde{\rho }}_{k^{\prime }k}=\text{sign}\left({\tilde{b}}_{k^{\prime }}^{\left(k\right)}\right)\sqrt{{\tilde{b}}_{k^{\prime }}^{\left(k\right)}{\tilde{b}}_{k}^{\left(k^{\prime }\right)}}$ if ${\tilde{b}}_{k^{\prime }}^{\left(k\right)}{\tilde{b}}_{k}^{\left(k^{\prime }\right)}>0$ and 0 otherwise, and ${\tilde{\pi }}_{k^{\prime }k}=-1$, ${\tilde{\rho }}_{k^{\prime }k}$, 1 if ${\tilde{\rho }}_{k^{\prime }k}$ is below −1, in the interval [−1,1], above 1, respectively. Accordingly, ${\tilde{w}}_{k^{\prime }k}=\left|{\tilde{\pi }}_{k^{\prime }k}\right|$ if $k^{\prime }\neq k$, 0 if $k^{\prime }=k$.

### 2.3 Adaptive estimation and NetARD

In the adaptive estimation approach, the hyper-parameters within Θ serve to regularize $\tilde{W}=\left({\tilde{w}}_{k^{\prime }k}\right)$ via regularizing ${\tilde{b}}^{\left(k\right)}$ (k=1,⋯,p) generated by the pARD algorithm. Let $s_{\beta }$, $r_{\beta }$ be fixed. This approach involves updating $\tilde{W}$ and fine-tuning $\left(s^{\left(1\right)},r^{\left(1\right)},\cdots ,s^{\left(p\right)},r^{\left(p\right)}\right)$ alternatingly over multiple iterations. Let us assume that $\tilde{W}$ is computed in one iteration of this estimation process. For k=1,⋯,p, we compute ${\tilde{v}}_{k}=\frac{1}{p}\sum_{j=1}^{p}{\tilde{w}}_{jk}\in \left[0,1\right]$. In the subsequent iteration, we update $r^{\left(k\right)}$, $s^{\left(k\right)}$ according to the following rules:

(7) $$\left\{ \begin{array}{c} r_{\text{new}}^{\left(k\right)}={\left(\lambda {\left(\frac{1-\xi }{\varphi \left({\tilde{w}}_{k^{\prime }k}\right)}+\frac{\xi }{\varphi \left({\tilde{\upsilon }}_{k^{\prime }}\right)}\right)+\frac{1}{\sqrt{2}}|}_{k^{\prime }\neq k}\right)}^{T}\\ s_{\text{new}}^{\left(k\right)}=r_{\text{new}}^{\left(k\right)}\circ r_{\text{new}}^{\left(k\right)}\; \end{array}\right.\text{.}$$

Here, λ>0, ξ∈[0,1] are fixed constants, the function φ(t) is a strictly increasing function of t that maps [0,1] into (0,∞), and the symbol ∘ is the operation of element-wise product. With the elements of $s^{\left(k\right)}$ greater than $\frac{1}{2}$ for k=1,⋯,p, Algorithm pARD generates ${\tilde{b}}^{\left(1\right)},\cdots ,{\tilde{b}}^{\left(p\right)}$ using the updated Θ. Note that $r^{\left(k\right)}$, $\frac{s_{\beta }}{\;r_{\beta }}$ are the prior means of $\alpha^{\left(k\right)}$, β, respectively. Additionally, $\frac{{\tilde{\alpha }}^{\left(k\right)}}{\tilde{\beta }}$ is the regularization weight vector of ${\tilde{b}}^{\left(k\right)}$. Indeed, we can view $\frac{r^{\left(k\right)}\;r_{\beta }}{\;s_{\beta }}$ as a form of “prior” regularization weight vector. Roughly, when ${\tilde{w}}_{k^{\prime }k}$ decreases (respectively, increases) in an iteration, it encourages an increase (respectively, decrease) in the “prior” regularization constants $\frac{r_{k^{\prime }}^{\left(k\right)}r_{\beta }}{{\;s}_{\beta }}$, $\frac{r_{k}^{\left(k^{\prime }\right)}r_{\beta }}{{\;s}_{\beta }}$ of ${\tilde{b}}_{k}^{\left(k^{\prime }\right)}$, ${\tilde{b}}_{k^{\prime }}^{\left(k\right)}$, respectively. Consequently, this encouragement leads to a further decrease (respectively, increase) in ${\tilde{w}}_{k^{\prime }k}$ in the subsequent iteration. Likewise, the inclusion of ${\tilde{\upsilon }}_{k^{\prime }}$ in $r_{\text{new}}^{\left(k\right)}$ has an impact on the change in ${\tilde{\upsilon }}_{k^{\prime }}$ during an iteration. A decrease (or increase) in ${\tilde{\upsilon }}_{k^{\prime }}$ in one iteration can indeed encourage a further decrease (increase) of ${\tilde{\upsilon }}_{k^{\prime }}$ in the subsequent iteration. This iterative process, often described as “the strong get stronger and the weak get weaker” effectively results in estimates of $w_{k^{\prime }k}$ $\left(k^{\prime }\neq k\right)$ with notably non-uniform estimates of $w_{k}=\sum_{j=1}^{p}w_{jk}$ (k=1,⋯,p). Algorithm apARD, which performs the adaptive estimation to generate $\tilde{W}$, is described in Section A2 in the Appendix.

Let us consider the assumption represented by $G_{0}=\left(V,E_{0}\right)$. In this context, the edges included in $E_{0}$ indicate likely edges, while the absence of edges in $E_{0}$ suggests that these edges are likely to be missing in the prior knowledge of G. To implement the apARD in this scenario, we scale down ${\tilde{w}}_{k^{\prime }k}$ to $r{\tilde{w}}_{k^{\prime }k}$ for $\left(k^{\prime },k\right)\notin E_{0}$ in each iteration of the adaptive estimation process, where r∈[0,1) is the shrinkage constant. The modified apARD can generate $\tilde{W}$ in which ${\tilde{w}}_{k^{\prime }k}$ $\left(k^{\prime },k\right)\notin E_{0}$ decrease toward 0. The occurrence of ${\tilde{w}}_{k^{\prime }k}>0$ $\left(k^{\prime },k\right)\notin E_{0}$ diminishes as r decreases to 0*.* The modified apARD is utilized to infer GNs with pre-specified hub genes. In this scenario, we define $\left(k^{\prime },k\right)\in E_{0}$ if $k^{\prime }\neq k$ and at least one of genes $k^{\prime }$, k is a given hub gene.

Let $\tilde{G}=\left(V,\tilde{E}\right)$ be the inferred GN, where $\left(k^{\prime },k\right)\in \tilde{E}$ if ${\tilde{w}}_{k^{\prime }k}>0$ and $\left(k^{\prime },k\right)\notin \tilde{E}$ otherwise. Under $X∼N_{p}\left(0_{p\times 1},\Sigma \right)$, we utilize a modified regression algorithm (Algorithm 17.1) from Ref. [14] to compute the maximum likelihood (ML) estimate $\hat{\Omega }$ of Ω that maximizes the log-likelihood l(Ω)=lndet(Ω)−trace(SΩ), where $S=\frac{1}{N}X^{T}X\in R^{p\times p}$, over all possible Ω≻0 of $\tilde{G}$. Accordingly, W is re-estimated by $\hat{W}$, where ${\hat{w}}_{k^{\prime }k}=\left|{\hat{\pi }}_{k^{\prime }k}\right|$ if $k^{\prime }\neq k$, 0 if $k^{\prime }=k$, and ${\hat{\pi }}_{k^{\prime }k}$ is obtained by substituting ${\hat{\omega }}_{k^{\prime }k}$ into Eq. (1) for $\omega_{k^{\prime }k}$. We refer to this hybrid method for generating $\tilde{G}$ using the apARD and $\hat{W}$ using the ML estimation of Ω for $\tilde{G}$ as the NetARD. The NetARD_H_ is the NetARD implemented with the modified apARD under given hub genes.

### 2.4 Co-expression extension of GN

The co-expressed genes are often involved in the same biological processes. Let us assume that a predefined core GN associated with specific biological pathways is given. We propose the Co-expression Extension of GN (CEGN) to extend the core GN. Let $s_{k^{\prime }k}=\left|\text{Corr}\left(X^{\left(k^{\prime }\right)},X^{\left(k\right)}\right)\right|$ estimate the similarity between $X_{k^{\prime }}$, $X_{k}$. The dissimilarities or distances between $X_{k^{\prime }}$, $X_{k}$ are calculated as $1-s_{k^{\prime }k}$ for all $k^{\prime }\neq k$, and then analyzed using hierarchical cluster analysis [15,16] to construct the co-expressed gene cluster hierarchy. Typically, the results are visualized using a dendrogram. The co-expressed gene sets are derived from the generated dendrogram using tree cut techniques [[17], [18], [19], [20]]. We utilize the modified NetARD to infer the GN within each co-expressed gene set that includes at least one of the core GN genes, where the core GN genes serve as hub genes. This enables us to identify interactions between core GN genes and their co-expressed genes. The core GN is expanded by incorporating the inferred interacting co-expressed genes and the interactions between them.

The computational experiments for GN inference and analysis are conducted in the R environment [21] on a personal computer running the Windows operating system. The computational settings of apARD are described at the end of Section A2 in the Appendix. When hub nodes are given, we set ξ=0, r=0 for the modified apARD. The $\tilde{W}$ resulting from the (modified) apARD is further sparsified by resetting ${\tilde{w}}_{k^{\prime }k}=0$ for ${\tilde{w}}_{k^{\prime }k}<10^{-3}$. The hierarchical clustering analysis involved in the CEGN uses a chosen linkage method to generate the gene cluster hierarchies. Following this, the co-expressed gene clusters are derived from the resulting cluster hierarchy using the dynamic tree cut algorithm [20] implemented in the R package WGCNA [22]. Unless otherwise stated, the above computational settings remain unchanged.

## 3 Results

### 3.1 Simulated data

**Fig. 1:**
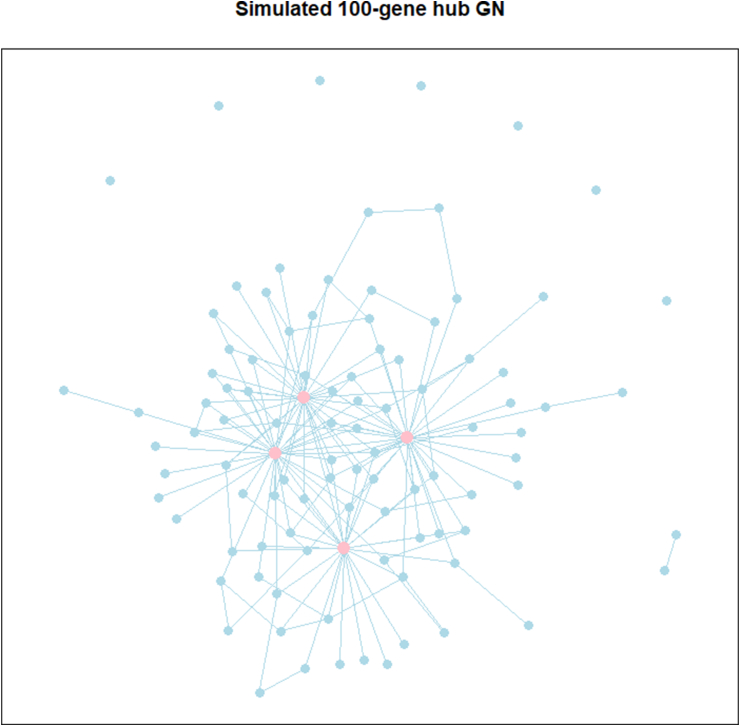
Graph of simulated 100-gene hub GN. The pink nodes represent hub genes that interact with more than 10 genes in the GN. (For interpretation of the references to color in this figure legend, the reader is referred to the Web version of this article.)

We perform simulation experiments to demonstrate the effectiveness of NetARD in inferring GNs with the hub genes (hub GNs). For these experiments, we use the R package hglasso [9] to simulate hub GNs with p=100 genes. Within each simulated graph, each hub node is connected to approximately an average of 32 nodes, while each non-hub node is connected to approximately an average of 2 nodes. The graph's sparsity level (SL) and hub level (HL) are defined as the fraction of missing edges among all possible edges and the connectivity degree centralization [23], respectively. Fig. 1 illustrates a simulated hub GN with 4 hub genes, exhibiting SL, HL ≈0.97, 0.33, respectively. In this diagram, the pink nodes represent the hub genes. For a simulated GN, we follow the procedure in Ref. [9] to generate the associated Ω≻0. More specifically, assume $A=\left(a_{k^{\prime }k}\right)\in R^{p\times p}$ is the adjacency matrix of the simulated graph G, where $a_{k^{\prime }k}=1$ if $\left(k^{\prime },k\right)\in E$, 0 otherwise. Ω is set equal to $A+\left(0.1-\Lambda_{\min}\left(A\right)I\right)$, where $\Lambda_{\min}\left(A\right)$ is the minimum eigenvalue of A and $I\in R^{p\times p}$ is the identity matrix. The simulated gene expression dataset comprises N=60 i.i.d. samples generated from $N_{p}\left(0_{p\times 1},\Omega^{-1}\right)$.

**Fig. 2:**
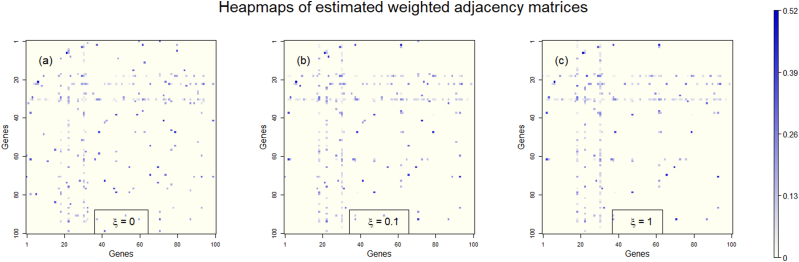
Heatmaps of estimated weighted adjacency matrices. The heatmaps display the estimated weighted adjacency matrices of hub GN obtained using the NetARD with ξ=0, 0.1, 1 in panels (a), (b), (c), respectively.

We begin by demonstrating the NetARD using the simulated gene expression data for the simulated GN in Fig. 1. The tuning parameters λ, ξ in the apARD control the level and type of regularization of $\tilde{W}$. For each ξ=0,0.1,1, we set λ to generate $\hat{G}$ with SL ≈0.97. The heatmaps of $\hat{W}$ generated by the NetARD are displayed in Fig. 2. The bluish dots of heatmap correspond to the edges of $\tilde{G}$, while the symmetrical pairs of horizontal and vertical stripes containing these bluish dots correspond to the hub genes. Notably, compared to panel (a), the bluish dots are more concentrated on specific stripes in the heatmaps of panels (b) and (c). The HLs of $\hat{G}$ associated with $\hat{W}$ are approximately 0.36, 0.46, 0.52, respectively. Let the genes be ranked based on the number of their interaction partners. The top 1–3 genes in each $\tilde{G}$ correspond to the actual hub genes of the hub GN.

We evaluate NetARD's performance in inferring 5 simulated hub GNs. These simulated GNs have varying numbers of hub genes, ranging from 2 to 6. Furthermore, we use same simulated gene expression data to compare NetARD's performance with other published graph estimation methods. The methods used for comparison are as below:

1. glasso [24]: It maximizes the regularized l(Ω) with the $L_{1}$-norm penalty to generate sparse estimates of Ω.
2. hglasso [9]: It maximizes the regularized l(Ω) with a convex penalty to produce the sparse estimates of Ω with the hub property.
3. DWLasso [8]: This method symmetrizes the estimates of $b^{\left(k\right)}$ (k=1,⋯,p) of node-wise weighted LASSO regressions with estimated normalized regularization weights. This produces sparse estimates of Ω with the hub property.

For the DWLasso, we re-estimate Ω by $\hat{\Omega }$, which maximizes l(Ω) subject to the estimate of G corresponding to the estimate of Ω produced by the DWLasso.

In the experiment, we set ξ=0.1 and use different values of λ for NetARD to generate $\hat{\Omega }$ with the SL of corresponding $\tilde{G}$ in a range near 1. Similarly, we set a grid of tuning parameters for each reference method to generate $\hat{\Omega }$. To evaluate the quality of $\hat{\Omega }$, we use the extended Bayesian information criterion (EBIC), calculated as ${\text{EBIC}}_{\gamma }\left(\hat{\Omega }\right)=-l\left(\hat{\Omega }\right)+\frac{\ln\;N+4\gamma \;\ln\;p}{N}\kappa$ [25]. Here, γ∈[0,1] and κ is the number of nonzero entries ${\hat{\omega }}_{k^{\prime }k}$ of $\hat{\Omega }$ with ${1\leq k}^{\prime }<k\leq p$. We set γ=0 for NetARD and 0.2 for reference methods. $\hat{W}$ is obtained from $\hat{\Omega }$ generating the lowest ${\text{EBIC}}_{\gamma }\left(\hat{\Omega }\right)$ among all generated $\hat{\Omega }$ for each method.

Several metrics are used to assess the accuracy of an estimated G. They include (Re), which is the percent of edges in G that are also present in the estimated G, fall-out (Fa), which is the percent of edges not found in G but present in the estimated G, and precision (Pr), which is the percent of edges in the estimated G that are also found in G. Given a computed $\hat{W}$ and a specific threshold, the estimated G consists of edges that correspond to edge weights in $\hat{W}$ that exceed the threshold. The receiver operating characteristic (ROC) curve plots Re against Fa of the estimated G as the threshold moves through the interval [0,1]. On the other hand, the precision-recall (PR) curve plots Pr against Re of the estimated G as the threshold moves through the interval [0,1]. To assess the efficiency of $\hat{W}$ in inferring G, we use the R package PRROC [26] to calculate the areas under the ROC curve (AUROC) and the PR curve (AUPR). The greater the AUROC and AUPR values are in interval [0,1], the more effective $\hat{W}$ is in inferring G.

**Fig. 3:**
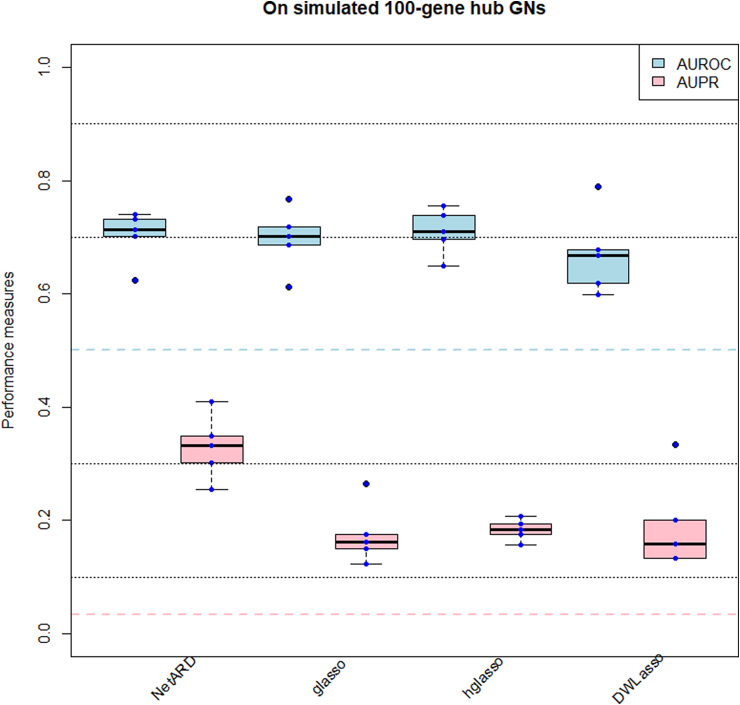
AUROCs, AUPRs and boxplots of AUROCs, AUPRs resulting from inference methods on 5 simulated 100-gene hub GNs. The color dots indicate the AUROCs, AUPRs yielded by GN inference methods on each of 5 simulated GNs. (For interpretation of the references to color in this figure legend, the reader is referred to the Web version of this article.)

Fig. 3 displays the AUROCs, AUPRs for each method in inferring 5100-gene hub GNs. The horizontal dashed lines in light blue and pink indicate the expected AUROC and AUPR of randomly generated W over the 5 GN inference experiments, respectively. These dashed lines serve as the baseline performance indicators. All compared methods yield AUROCs, AUPRs above the baseline levels. Table 1 presents the median values of AUROC, AUPR for $\hat{W}$, as well as the median values of SL, HL of estimated G corresponding to $\hat{W}$ generated by each method. When ranking the compared methods for sparse GN inference based on the median AUPR, it is observed that the NetARD performs best in this experiment.

**Table 1:** Median values of AUROC, AUPR, SL, HL resulting from GN inference methods on 5 simulated 100-gene hub GNs.

Methods	AUROC	AUPR	SL	HL
NetARD	0.71	0.33	0.98	0.37
glasso	0.70	0.16	0.98	0.37
hglasso	0.71	0.18	0.98	0.38
DWLasso	0.67	0.16	0.97	0.38

### 3.2 GNW in silico data

**Fig. 4:**
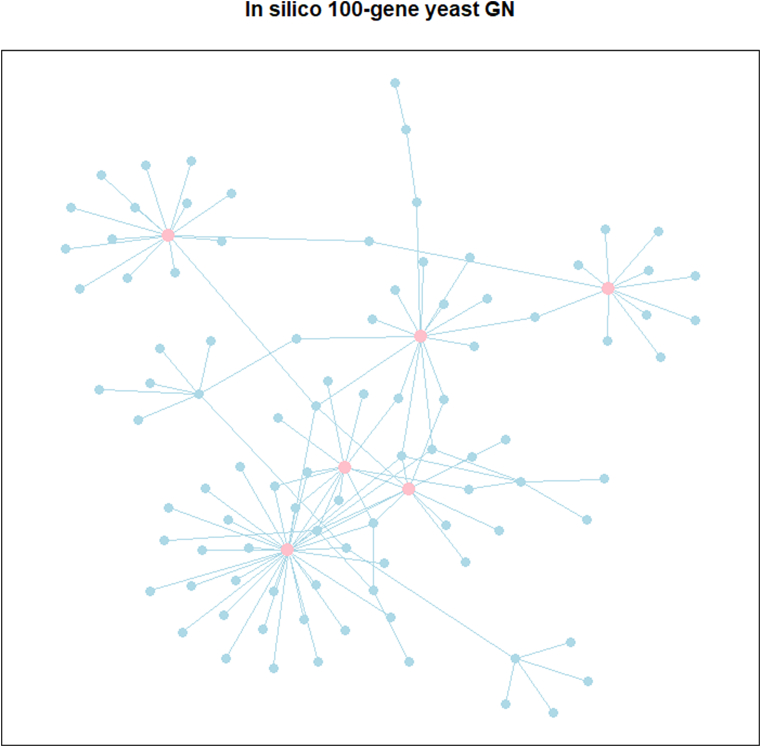
Graph of in silico 100-gene yeast GN. The pink nodes represent hub genes that interact with more than 5 genes in the GN. (For interpretation of the references to color in this figure legend, the reader is referred to the Web version of this article.)

The GeneNetWeaver (GNW) [27] is a software tool that generates in silico GNs by extracting subnetworks from real GNs and simulates gene expression data using a system of non-linear ordinary differential equations. The dataset gnw2000 in the R package grndata [28] includes an undirected yeast GN with 2000 genes and 2000 simulated steady-state samples of noise-free gene expression data of GN generated by GNW. For our study, we generate 5 test datasets, each of which includes a p=100-gene connected subnetwork extracted from the yeast GN and N=60 samples of gene expression data extracted from the first 60 samples of gene expression data in gnw2000 dataset. To simulate observation errors, we add Gaussian noise generated using the standard deviation of 0.01 to the extracted data. Fig. 4 illustrates one of the generated 100-gene yeast GN that contains 119 edges, with pink nodes representing hub genes that connect to more than 5 other genes.

**Fig. 5:**
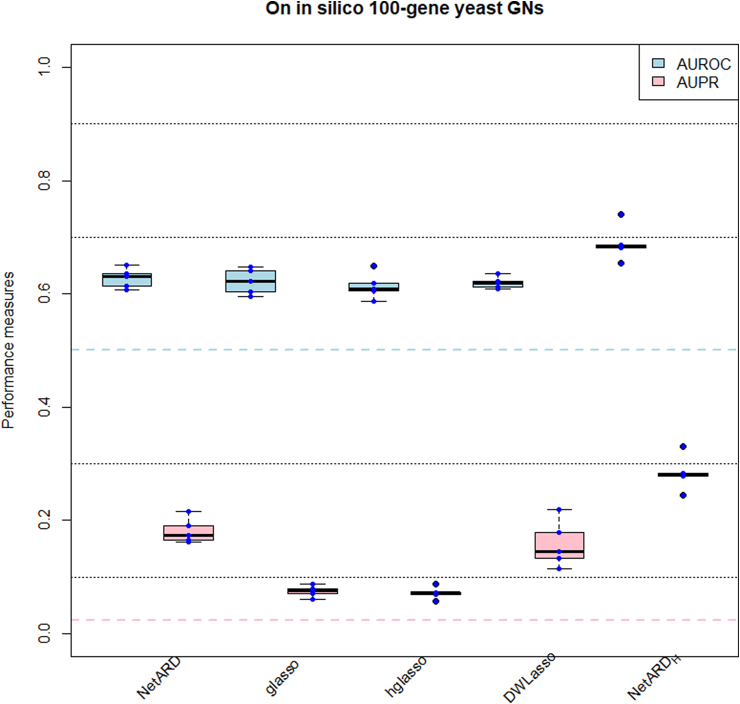
AUROCs, AUPRs and boxplots of AUROCs, AUPRs resulting from GN inference methods on 5 in silico 100-gene yeast GNs.

In the experiments for GN inference, we first standardize the expression data for each gene in a gene expression dataset. This is achieved by centering the mean to 0 and scaling the standard deviation to 1. The GN inference methods are then applied to these standardized gene expression datasets using similar computational settings as in previous experiments. We also implement the NetARD_H_ on data, considering the provided hub genes. Fig. 5 displays the AUROCs, AUPRs and boxplots for each method used to infer 5100-gene yeast GNs. Table 2 presents the median values of AUROC, AUPR of $\hat{W}$ and the median values of SL, HL of estimates of G yielded by each method. Overall, the NetARD outperforms the reference methods in terms of generating higher AUPRs. The performance of NetARD_H_ exhibits significant improvement compared to that of NetARD.

**Table 2:** Median values of AUROC, AUPR, SL, HL resulting from GN inference methods on 5 in silico 100-gene yeast GNs.

Methods	AUROC	AUPR	SL	HL
NetARD	0.63	0.17	0.96	0.11
glasso	0.62	0.08	0.97	0.08
hglasso	0.61	0.07	0.98	0.08
DWLasso	0.62	0.15	0.95	0.07
NetARD_H_	0.68	0.28	0.96	0.30

### 3.3 *E. coli* microarray data

The Many Microbe Microarrays Database (M^3D^) [29] provides gene expression datasets measured using Affymetrix microarrays. From this database, we select the *E. coli* dataset, which includes 466 samples of expression data for 4297 genes. To acquire gene and transcription factor (TF) annotations, as well as experimentally validated regulatory relationships between TFs and genes in *E. coli* K-12, we utilize the R package regutools [30] to access datasets from RegulonDB [31].

To prepare the data for analysis, genes lacking gene symbol identifiers are excluded from the microarray dataset. The expression data of the remaining 4212 genes are log-transformed and then standardized. To construct the gold standard GN, we utilize the gene-gene regulation dataset, which includes 4405 regulatory relationships involving 201 TF-encoding gene or multi-gene regulators and 1842 target genes. We split multi-gene regulators into individual gene regulators. Genes not present in the measured genes for analysis, along with regulations involving missing regulators or target genes, are excluded. Self-regulations are omitted, and ultimately, regulatory directions are disregarded. The resulting gold standard GN includes 1887 genes and 4791 gene-gene interactions. Moreover, we utilize the TF-TF regulation dataset from the database, which includes 177 TFs and 449 TF-TF regulatory relationships, to extract interactions between TF genes from the gold standard GN. The resulting TF gene GN includes 154 TF genes and 333 gene-gene interactions. We observe that the gold standard GN exhibits a relatively low coverage of gene-gene interactions between the 4212 genes used for analysis.

**Fig. 6:**
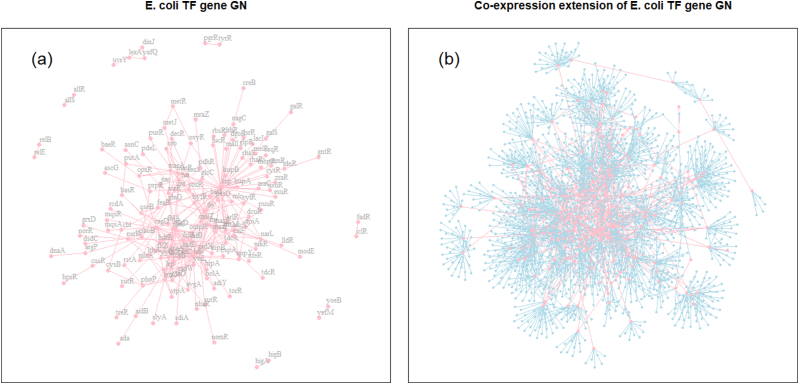
(a) GN of TF genes of *E. coli* (b) Co-expression extension of GN of TF genes of *E. coli*.

To evaluate the performance of GNCE, we apply it to extend the TF gene GN using the processed microarray data. By employing the “Ward.D2” linkage for the hierarchical clustering and applying the dynamic tree cut algorithm [20] with the minimum cluster size set to 7 to the resulting cluster hierarchy, we obtain 148 co-expressed gene clusters and 1 noise gene cluster. We have utilized the largest minimum cluster size in the dynamic tree cut algorithm so that the noise gene cluster does not contain any TF genes. We identify and select 79 co-expressed gene sets, each of which includes TF genes. The sizes of these sets range from 7 to 117 genes. A total of 264 gene-gene interactions within the gold standard GN fall within the selected co-expressed gene sets. Subsequently, the NetARD_H_ is utilized to infer the GN within each of the 79 co-expressed gene sets, where the included TF genes serve as hub genes. As a result, in the 2006 inferred gene-gene interactions, 203 of them match the gold standard gene-gene interactions with Re≈0.77 and Pr≈0.10. The limited coverage of gene-gene interactions in the gold standard GN may contribute to the significantly lower Pr compared to the median Pr≈0.28 induced by the NetARD_H_. Merging the TF gene GN and the inferred gene-gene interactions results in the extension of TF GN that comprises 1630 genes. Out of 2327 edges of this inferred extension, 524 edges correspond to the edges within the gold standard GN. Fig. 6 (a) illustrates the TF gene GN and (b) the extension of TF gene GN.

### 3.4 Colon cancer RNAseq data

The Cancer Genome Atlas (TCGA) Project is a useful resource for obtaining human cancer gene expression data. In our study, we use the COAD RNA sequencing (RNAseq) dataset, which pertains to colon adenocarcinoma and is available through the R package RTCGA.rnaseq [32]. The dataset provides normalized RNAseq expression data of 285 cancer tissue and 41 matched normal tissue samples, encompassing 20531 human genes. To acquire the predefined gene-gene interactions involved in colorectal cancer, we refer to the pathway map hsa05210 stored in the Kyoto Encyclopedia of Genes and Genomes (KEGG) [33]. This pathway map outlines the molecular interactions during the development of human colorectal cancer. To obtain the GN associated with this pathway map, we use the R package KEGGgraph [34] to parse the hsa05210 file that is in extensible markup language (XML) format. As a result, we generate a KEGG colorectal cancer GN comprising 86 genes and 149 edges. Here, the regulatory directions between genes are not considered.

To prepare the data for analysis, we take a few steps of data preprocessing. First, genes with missing values are excluded. In addition, genes with duplicated gene symbol identifiers or genes that are not annotated by R package org.Hs.eg.db [35] are excluded. Furthermore, genes with expression levels lower than 0.1 in 95% of the samples are excluded from the dataset. The expression data of the remaining 16234 genes are first log-transformed and then standardized. We then use the R package limma [36] to conduct a differential expression analysis using the standardized log-transformed gene expression data. This package utilizes robust statistical methods in a linear modeling framework to test whether the gene expression levels are significantly different in cancer and normal tissues and control for multiple testing. From this analysis, a total of 11677 genes are identified as differentially expressed (DE) across cancer and normal tissue samples, with their false discovery rate (FDR) adjusted p-values below 0.05. On the other hand, 15 KEGG colorectal cancer GN genes are identified as non-DE genes.

**Fig. 7:**
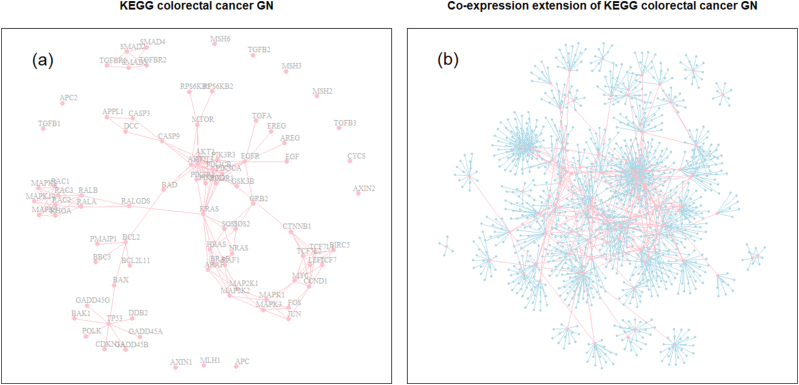
(a) KEGG colorectal cancer GN (b) Co-expression extension of KEGG colorectal cancer GN.

We apply the GNCE to the standardized log-transformed expression data for 11692 DE and KEGG colorectal cancer GN genes obtained from 285 cancer tissue samples. By employing the “complete” linkage for the hierarchical clustering and applying the dynamic tree cut algorithm with the minimum cluster size set to 10, we obtain 559 co-expression gene clusters and 1 noise gene cluster. Except for 1 gene (SAMAD2) included in the noise gene cluster, the remaining 85 KEGG colorectal cancer GN genes are distributed across 74 co-expressed gene clusters. The NetARD_H_ with r=0.2 is utilized to infer the GN within each of these 74 co-expressed gene sets, where the included KEGG colorectal cancer GN genes serve as hub genes. As a result, we identify 1144 interactions between hub genes and their co-expressed genes. Merging the KEGG colorectal cancer gene GN and the inferred gene-gene interactions results in the extended GN that contains 1269 genes and 4386 edges. Fig. 7 (a) illustrates the KEGG colorectal cancer gene GN and (b) the extension of KEGG colorectal cancer gene GN.

### 3.5 Colorectal cancer-related Gene Ontology terms

We analyze the inferred GN presented in Fig. 7 (b) to discover Gene Ontology (GO) terms related to biological processes (BPs) that play an important role in the context of colorectal cancer progression. We first employ GO enrichment analysis (GOEA) to identify BP GO terms significantly enriched in each co-expressed gene set within the inferred GN. In this analysis, we use the gene set of inferred GN as the background gene set. For each GO term, the associated gene set comprises genes from the background gene set that are annotated by that particular GO term. The strength of association between a GO term and a co-expressed gene set within the inferred GN is assessed by evaluating the strength of association between the gene set associated with the GO term and the co-expressed gene set. We employ the R package topGO [37] to compute the p-value of the Fisher exact test, which quantifies the likelihood of observing such an association through random sampling. BP GO terms with their FDR adjusted p-values lower than a specified level are considered significantly enriched in the co-expressed gene set.

Subsequently, we proceed to identify the GO terms that are significantly associated with the inferred GN from the previously identified enriched BP GO terms. Here, a GO term is considered associated with the inferred GN if the genes of its associated gene set distribute closely in the inferred GN. We utilize the Knet function [38], akin to Ripley's K-function, to examine the distribution of gene set within the inferred GN. A higher value of the area under the Knet function (AUK) indicates a greater degree of clustering of gene set within the inferred GN. A GO term is associated with the inferred GN if the AUK of its associated gene set significantly surpasses that of any randomly selected gene set of the same size. We employ the R package SANTA [38], where the shortest-path distance is utilized to quantify the distance between two genes within the GN, to compute the p-value of permutation test, by comparing the AUK of the GO term gene to the AUKs of randomly generated gene sets of the same size as the GO term gene set, for each GO term of interest. BP GO terms with their FDR adjusted p-values lower than a specified level are considered significantly associated with the inferred GN.

In the GO term analysis experiment, we set the significance level of the FDR-adjusted p-value for Fisher exact tests in GOEA at 0.05. We identify 891 BP GO terms that exhibit significant enrichment within 29 co-expressed gene sets. We choose from these BP GO terms whose associated gene sets contain 5 or more genes for subsequent analysis. Employing a significance level of FDR-adjusted p-value at 0.1 for permutation tests, we identify 40 GO terms, which exhibit significant enrichment within 11 co-expressed gene sets, significantly associated with the inferred GN. We present the results of our GO term analysis pertaining to a specific co-expressed gene set as follows.

**Fig. 8:**
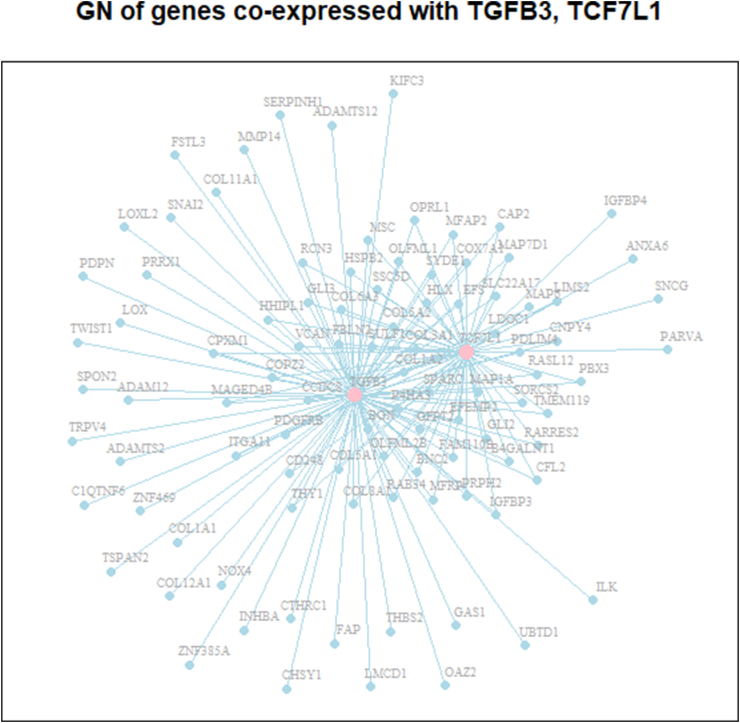
Graph of 103 co-expressed gene subnetwork of the inferred GN in Fig. 8 (b). The pink nodes represent hub genes TGFB3, TCF7L1 in Fig. 7 (a). (For interpretation of the references to color in this figure legend, the reader is referred to the Web version of this article.)

**Fig. 9:**
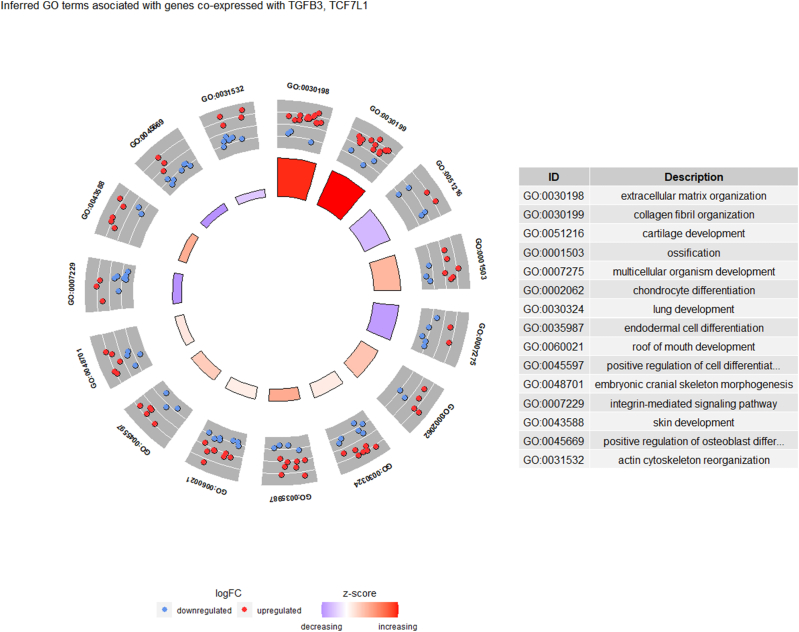
GO plot of identified BP GO terms associated with colorectal cancer. In this plot, red and blue dots represent the upregulated (logFC>0) and downregulated (logFC<0) GO term genes on the inferred GN in Fig. 7 (b), respectively. Here, the logFC represents the difference of averages of log2-transformed gene expression data over cancer and normal tissue samples. The z-score of gene set is calculated as $\frac{u-d}{\sqrt{c}}$, where u is the number of upregulated genes, d is the number of downregulated genes, and c is the total number of genes of gene set. (For interpretation of the references to color in this figure legend, the reader is referred to the Web version of this article.)

Fig. 8 illustrates the largest subnetwork consisting of 103 co-expressed genes within the inferred GN shown in Fig. 7 (b). In this diagram, the pink nodes represent hub genes, including TGFB3 (Transforming Growth Factor Beta 3) and TCF7L1 (Transcription Factor 7-like 1), both included in the KEGG colorectal cancer GN. Among the 92 BP GO terms significantly enriched in genes in Fig. 8, there are 38 GO terms associated with gene sets containing 5 or more genes. Fig. 9 depicts the GO plot for the final 15 BP GO terms that are significantly associated with the inferred GN. These identified GO terms are related to the organization of extracellular matrix components (GO:0030198, GO:0030199), the development and differentiation of connective tissues (GO:0051216, GO:0001503, GO:0002062, GO:0048701, GO:0045669) and skins (GO:0043588), the development and differentiation of organs and tissues within a multicellular organism (GO:0007275, GO:0030324, GO:0035987, GO:0060021), as well as cell signaling (GO:0007229), regulation of cell differentiation (GO:0045597), and the reorganization of the actin cytoskeleton (GO:0031532). These GO terms provide informative annotations for the co-expressed genes associated with cancer-related BPs.

**Fig. 10:**
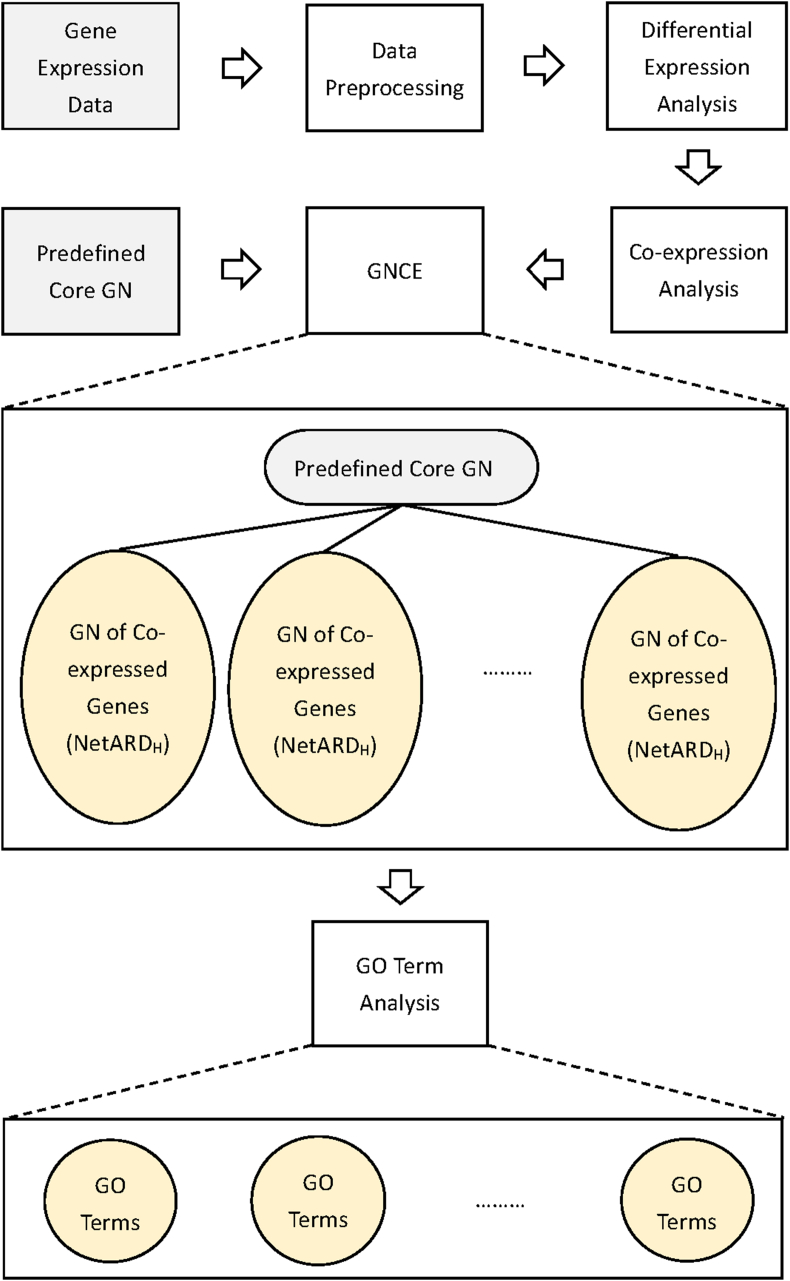
Workflow of the proposed method for identifying cancer-related GO terms using gene expression data and predefined core GN specific to cancer.

Fig. 10 illustrates the workflow of the proposed method for identifying cancer-related GO terms using gene expression data and predefined core GN of cancer.

## 4 Conclusion

According to the AUPR, which provides a more insightful measure of sparse GN inference performance, the NetARD outperforms the reference GN inference methods when applied to simulated hub GNs and in silico yeast GNs. When provided with hub genes, the NetARD significantly improves the inference accuracy on in silico yeast GNs. The GNCE, which integrates co-expression analysis and the NetARD, retrieves the majority of interactions between TF genes and their co-expressed genes of *E. coli*. The GNCE enables the prediction of GO terms associated the colorectal cancer using combined information from curated core cancer GN and gene expression data.

## 5 Discussion

The proposed NetARD utilizes the apARD to infer the GN and then re-estimates the non-zero partial correlations between genes using the GGM. It is important to note that gene expression data can frequently display non-normally distributed characteristics, and the gene expression profiles can exhibit complex nonlinear correlations. Partial correlation or GGM-based graph estimation methods primarily target the capture of direct linear dependencies between (log-transformed) gene expression levels. Consequently, these methods may encounter difficulties in identifying GNs, particularly as the size of GN grows larger.

It is known that multiple genes frequently collaborate and exhibit co-expression during cellular processes. The GNCE identifies interactions between predefined core GN genes and their co-expressed genes using the NetARD, achieved through the clustering of genes into small-scale co-expressed gene sets. In our experiments, by controlling the minimum cluster size in the dynamic tree cut algorithm, the resulting co-expressed gene sets typically contain below 120 genes. Smaller co-expressed gene set sizes allow the NetARD to efficiently infer GNs with shorter computational time. However, this GNCE does not capture the interactions between non-co-expressed genes. To identify GO terms associated with the progression of colorectal cancer, we employ the GN inferred by GNCE, incorporating KEGG colorectal cancer core GN and gene expression data. Our approach is based on the fundamental assumption that GNs consisting of genes displaying co-expression patterns and interacting with those involved in the curated cancer pathway primarily contribute to the coordination of cellular processes.

Finally, additive models extend linear regressions by incorporating nonlinear component functions, such as spline functions, to accommodate the nonlinear interactions between predictor variables and the response variable. One of our future research interests involves the inference of GNs with biologically meaningful topological properties using additive models of gene expression. The GNs inferred in this context are expected to yield novel insights into the interactions among genes that coordinate cellular processes in both healthy and diseased states.

## CRediT authorship contribution statement

**Chi-Kan Chen:** Writing – original draft, Software, Methodology, Investigation, Formal analysis, Conceptualization.

## Declaration of competing interest

The authors declare that they have no known competing financial interests or personal relationships that could have appeared to influence the work reported in this paper.

## Appendix

A.1 p-node ARD.

From the normality assumptions of $P_{k}\left(\varepsilon_{k}|b^{\left(k\right)},\beta \right)$, $P_{k}\left(b^{\left(k\right)}|\alpha^{\left(k\right)}\right)$ and the application of conditional probability, we can express $P_{k}\left(b^{\left(k\right)}|X,\alpha^{\left(k\right)},\beta \right)$ in Eq (4) as

(A1) $${\left(2\pi \right)}^{-\;\frac{p-1}{2}}{\left|{\tilde{S}}_{b^{\left(k\right)}}\right|}^{-\frac{1}{2}}\;\exp\left\{-\frac{1}{2}{\left(b^{\left(k\right)}-{\tilde{b}}^{\left(k\right)}\right)}^{T}{\tilde{S}}_{b^{\left(k\right)}}^{-1}\left(b^{\left(k\right)}-{\tilde{b}}^{\left(k\right)}\right)\right\}\text{,}$$

where ${\tilde{S}}_{b^{\left(k\right)}}={\left(\beta {X^{\backslash k}}^{T}X^{\backslash k}+D_{\alpha^{\left(k\right)}}\right)}^{-1}$, ${\tilde{b}}^{\left(k\right)}=\beta {\tilde{S}}_{b^{\left(k\right)}}{X^{\backslash k}}^{T}X^{\left(k\right)}$. Thus, $P_{k}\left(b^{\left(k\right)}|X,\alpha^{\left(k\right)},\beta \right)$ follows $N_{p-1}\left({\tilde{b}}^{\left(k\right)},{\tilde{S}}_{b^{\left(k\right)}}\right)$. Moreover, we can express $L_{k}\left(\alpha^{\left(k\right)},\beta |X\right)$ in Eq (4) as

(A2) $${\left(2\pi \right)}^{-\;\frac{N}{2}}\beta^{\frac{N}{2}}{\left|D_{\alpha^{\left(k\right)}}\right|}^{\frac{1}{2}}{\left|{\tilde{S}}_{b^{\left(k\right)}}\right|}^{\frac{1}{2}}\;\exp\left\{-\frac{\beta }{2}{\left\|X^{\left(k\right)}-X^{\backslash k}{\tilde{b}}^{\left(k\right)}\right\|}_{2}^{2}-\frac{1}{2}{{\tilde{b}}^{\left(k\right)}}^{T}D_{\alpha^{\left(k\right)}}{\tilde{b}}^{\left(k\right)}\right\}\text{.}$$

Using Eq. (A2), for $k^{\prime }\neq k$, we derivative the partial derivatives $\frac{\partial }{\partial \alpha_{k^{\prime }}^{\left(k\right)}}\ln\;L_{k}\left(\alpha^{\left(k\right)},\beta |X\right)$ as

(A3) $$\frac{1}{2\alpha_{k^{\prime }}^{\left(k\right)}}-\frac{1}{2}\left\{ \begin{array}{c} {\tilde{S}}_{b^{\left(k\right)}}\left[k^{\prime }-1,k^{\prime }-1\right]\;\text{if}\;k<k^{\prime }\\ {\tilde{S}}_{b^{\left(k\right)}}\left[k^{\prime },k^{\prime }\right]\;\text{if}\;k>k^{\prime }\; \end{array}\right.-\frac{1}{2}{{\tilde{b}}_{k^{\prime }}^{\left(k\right)}}^{2}$$

and $\frac{\partial }{\partial \beta }\ln\;L_{k}\left(\alpha^{\left(k\right)},\beta |X\right)$ as

(A4) $$\frac{N}{2\beta }-\frac{1}{2}\text{trace}\left(\;{\tilde{S}}_{b^{\left(k\right)}}{X^{\backslash k}}^{T}X^{\backslash k}\right)-\frac{1}{2}{\left\|X^{\left(k\right)}-X^{\backslash k}{\tilde{b}}^{\left(k\right)}\right\|}_{2}^{2}.$$

Likewise, for log-Gamma priors, $\frac{\partial }{\partial \alpha_{k^{\prime }}^{\left(k\right)}}\ln\;P_{k}\left(\alpha^{\left(k\right)}|\theta^{\left(k\right)}\right)=\frac{s_{k^{\prime }}^{\left(k\right)}-1}{\alpha_{k^{\prime }}^{\left(k\right)}}-r_{k^{\prime }}^{\left(k\right)}$, $\frac{\partial }{\partial \beta }\ln\;P\left(\beta |\theta_{\beta }\right)=\frac{s_{\beta }-1}{\beta }-r_{\beta }$. By solving $\frac{\partial }{\partial \alpha_{k^{\prime }}^{\left(k\right)}}f\left(\alpha^{\left(1\right)},\cdots ,\alpha^{\left(p\right)},\beta \right)=0$, we obtain the maximizer of $f\left(\alpha^{\left(1\right)},\cdots ,\alpha^{\left(p\right)},\beta \right)$ in Eq. (5) with $\alpha^{\left(k^{\prime }\right)}$ $\left(k^{\prime }\neq k\right)$, β fixed as

(A5) $$\alpha^{\left(k\right)}=\frac{2s^{\left(k\right)}{-1}_{\left(p-1\right)\times 1}}{\text{diag}\left({\tilde{S}}_{b^{\left(k\right)}}\right)+{\tilde{b}}^{\left(k\right)}\circ {\tilde{b}}^{\left(k\right)}+2r^{\left(k\right)}}\text{,}$$

where $\text{diag}\left({\tilde{S}}_{b^{\left(k\right)}}\right)$ is the diagonal vector of ${\tilde{S}}_{b^{\left(k\right)}}$ and the vector division is the element-wise division. By solving $\frac{\partial }{\partial \beta }f\left(\alpha^{\left(1\right)},\cdots ,\alpha^{\left(p\right)},\beta \right)=0$), we obtain the maximizer of $f\left(\alpha^{\left(1\right)},\cdots ,\alpha^{\left(p\right)},\beta \right)$ with $\alpha^{\left(k\right)}$ (1,⋯,p) fixed as

(A6) $$\beta =\frac{N-2+2s_{\beta }}{\frac{1}{p}\sum_{k=1}^{p}\left\{\text{trace}\left(\;{\tilde{S}}_{b^{\left(k\right)}}{X^{\backslash k}}^{T}X^{\backslash k}\right)+{\left\|X^{\left(k\right)}-X^{\backslash k}{\tilde{b}}^{\left(k\right)}\right\|}_{2}^{2}\right\}+2r_{\beta }}\text{.}$$

Assume Θ with $s_{k^{\prime }}^{\left(k\right)}>\frac{1}{2}$ $\left(k^{\prime }\neq k\right)$, and the initial values of $\alpha^{\left(k\right)}$ (k=1,⋯,p), β are specified. Algorithm pARD that computes ${\tilde{S}}_{b^{\left(k\right)}}$, ${\tilde{b}}^{\left(k\right)}$ under $\alpha^{\left(k\right)}$, β, and updates $\alpha^{\left(k\right)}$, β using Eq. (A5), (A6), respectively, iteratively to obtain the optimal ${\tilde{b}}^{\left(k\right)}$, ${\tilde{\alpha }}^{\left(k\right)}$ (k=1,⋯,p), $\tilde{\beta }$ is as follows.

Algorithm pARD

repeat

for k=1,⋯,p do

$${{C_{k}\leftarrow X}^{\backslash k}}^{T}X^{\backslash k}$$

$$S_{b^{\left(k\right)}}\leftarrow {\left(\beta C_{k}+D_{\alpha^{\left(k\right)}}\right)}^{-1}$$

$$b^{\left(k\right)}\leftarrow \beta S_{b^{\left(k\right)}}{X^{\backslash k}}^{T}X^{\left(k\right)}$$

$$\alpha^{\left(k\right)}\leftarrow \frac{2s^{\left(k\right)}{-1}_{\left(p-1\right)\times 1}}{\text{diag}\left(S_{b^{\left(k\right)}}\right)+{\tilde{b}}^{\left(k\right)}\circ {\tilde{b}}^{\left(k\right)}+2r^{\left(k\right)}}$$

end for

$$\beta \leftarrow \frac{N-2+2s_{\beta }}{\frac{1}{p}\sum_{k=1}^{p}\left\{\text{trace}\left(\;S_{b^{\left(k\right)}}C_{k}\right)+{\left\|X^{\left(k\right)}-X^{\backslash k}b^{\left(k\right)}\right\|}_{2}^{2}\right\}+2r_{\beta }}$$

if $f\left(\alpha^{\left(1\right)},\cdots ,\alpha^{\left(p\right)},\beta \right)$ ceases to increase then break

end

$$\text{return}\;\tilde{B}=\left(b^{\left(1\right)},\cdots ,b^{\left(p\right)}\right),\tilde{Α}=\left(\alpha^{\left(1\right)},\cdots ,\alpha^{\left(p\right)}\right),\tilde{\beta }=\beta$$

A2. Adaptive p-node ARD.

Assume the parameters λ, ξ, $s_{\beta }$, $r_{\beta }$ are fixed. Assume the initial W and the initial values of $\alpha^{\left(k\right)}$ (k=1,⋯,p), β for Algorithm pARD are specified. Denote by Φ the transformation of $\tilde{B}=\left({\tilde{b}}^{\left(1\right)},\cdots ,{\tilde{b}}^{\left(p\right)}\right)$ to $\tilde{W}$ as described in Section 2.3. Algorithm apARD solves $\tilde{W}$ with the adaptively tuned $\left(s^{\left(k\right)},r^{\left(k\right)}\right)$ (k=1,⋯,p) is as follows.

Algorithm apARD

repeat

for k=1,⋯,p do

$$r^{\left(k\right)}\leftarrow {\left(\lambda {\left(\frac{1-\xi }{\varphi \left({\tilde{w}}_{k^{\prime }k}\right)}+\frac{\xi }{\varphi \left(\frac{1}{p}\sum_{j=1}^{p}{\tilde{w}}_{jk^{\prime }}\right)}\right)+\frac{1}{\sqrt{2}}|}_{k^{\prime }\neq k}\right)}^{T}$$

$$s^{\left(k\right)}\leftarrow r^{\left(k\right)}\circ r^{\left(k\right)}$$

end for

$$B\leftarrow \text{pARD}\left(X;\Theta \right)\;{|}_{\tilde{B}}$$

W←Φ(B)

if W ceases to change then break

end repeat

return $\tilde{W}=W$, Θ.

In our computational experiments, we use a fix the Gamma prior over β with $s_{\beta }=1$, $r_{\beta }=10^{-3}$ to approximate the flat prior. We define $\varphi \left(t\right)=\frac{t+10^{-3}}{1-t+10^{-3}}$, t∈[0,1]. To initialize the apARD, we set the initial W with $k^{\prime },k$ entry of $w_{k^{\prime }k}=\left|\text{Cor}\left(X^{\left(k^{\prime }\right)},X^{\left(k\right)}\right)\right|$ if $k^{\prime }\neq k$, 0 if $k^{\prime }=k$, and the initial $\alpha^{\left(k\right)}=r^{\left(k\right)}$ (k=1,⋯,p), β=1. The maximum iteration numbers of apARD, pARD are 10, 50, respectively.
